# QUASUS: A Tool for Measuring the Parents’ School Satisfaction

**DOI:** 10.3389/fpsyg.2019.00013

**Published:** 2019-01-22

**Authors:** Piergiorgio Mossi, Emanuela Ingusci, Marco Tonti, Sergio Salvatore

**Affiliations:** Department of History, Society and Human Studies, University of Salento, Lecce, Italy

**Keywords:** QUASUS, customer satisfaction in educational contexts, parent school satisfaction, scale development, prosumership

## Abstract

No validated instruments for assessing school users’ satisfaction are available. This paper means to contribute to address this lack. It outlines a new instrument of measurement of school users’ satisfaction – QUASUS (QUestionnaire for the Analysis of the School User’s Satisfaction). The main peculiarity of QUASUS lies in the fact that it pays specific attention to the dimension of prosumership, namely the view of the client-service co-constructive relation as a constitutive component of the service’s construction and delivery. The study reports the output of an initial validation study of the instrument. Based on two samples of parents (*N* = 2802 and *N* = 1365) from Italian schools, analyses provided support to the hypotheses tested: QUASUS proved to be characterized by a good level of reliability (HP1); is able to detect the component comprising the school users’ satisfaction (HP2); proves a global connotation of the experience of the school by a unidimensional measure of the overall satisfaction (HP3), associated significantly with the prosumership (HP4).

## Introduction

The notion of Customer Satisfaction was developed within the Quality Management framework, with the aim of evaluating the performance of business organizations in terms of their capacity of fulfil clients’ expectations (Anderson et al., 1994; Oliver, 2010). From that field, the concept spread to the field of Public Administration (Brown and Coulter, 1983; Van Ryzin, 2004; Charbonneau and Van Ryzin, 2012), though rather slowly and partially, due to several theoretical and methodological concerns as to the generalizability of the approach to organizations that do not have business purposes (Stipak, 1979, Charbonneau and Van Ryzin, 2012; Olsen, 2015). In more recent years, concepts and measures of customer satisfaction have started to be implemented within the educational context too (Salisbury et al., 1997; Johnson and Kattman, 2003; Friedman et al., 2007), also in this case with some criticism as to their validity and consistency with the particular nature of education institutions (Bruni and Zamagni, 2007; Argandoña, 2011). So far, most of the efforts have focused on the higher education context (Aldridge and Rowley, 1998; Munteanu et al., 2010), while fewer studies (Griffith, 1997, 1998, 2001; Fantuzzo et al., 2006) concern public primary and high schools (Salisbury et al., 1997; Bond and King, 2003; Skallerud, 2011; Charbonneau and Van Ryzin, 2012).

One can identify three main focuses of the latter line of studies. First, several studies deal with the relation between perceived school quality and ethnic identity (Griffith, 1998; Kleitz et al., 2000; Weiher and Tedin, 2002; Thompson, 2003; Tedin and Weiher, 2004; Friedman et al., 2006). These studies were carried out in the US school context, where ethnic differences are a relevant facet that school management has to address. In general, these studies analyse whether ethnic groups are associated with different levels of satisfaction and participation in school activities. Friedman et al. (2006), for instance, examined the differences in school satisfaction among four ethnic groups, on a very large sample of parents. They found that factors like facilities and equipment were more important for some ethnic groups, whereas no difference between groups was detected on the school budget and the effectiveness of the teacher. A second main focus concerns studies that analyse whether and to what extent the parent’s choice of school is a factor that affects customer satisfaction (Bonstingl, 1992). This focus is specifically relevant in countries where schools are in competition with each other in order to be chosen by parents (Griffith, 1998; Hausman and Goldring, 2000; Kelly, 2005; Fantuzzo et al., 2006; Li and Hung, 2009; Skallerud, 2011). For instance, Hausman and Goldring (2000) carried out a study to assess the selection of magnet schools, a public school that typically focuses on individually themed curricula. They found that parents who report choosing for reasons of value (such as the teaching style) and academic reasons (such as special programs) reported higher levels of satisfaction than those who chose for reasons of convenience (such as closeness of school to their workplace). Third, some studies deal with satisfaction in the context of the issue of how to promote parents’ involvement and cooperation with school. An example of this kind of studies is provided by Falbo et al. (2003), who found low correlation between parental involvement in school activities and satisfaction. The last focus of investigation is connected with a broader interest in understanding the mechanism underpinning the citizens’ engagement with institutions, with respect to which the parent-school interaction represents a prototypic instance. These studies suggest caution with respect to an immediate generalization of the concept of customer satisfaction from the private, business context to the institutional context – and more specifically, schools. Indeed, as findings from Jacobsen et al. (2014) suggest, users of school services do not regard themselves necessarily as clients. Instead, they may consider the school as committed to meeting standards imposed by the institutional system; consequently, parents may not feel involved in a relation of reciprocity motivating them to respond with cooperation to the school effort to improve quality.

Taken as a whole, these studies have had the merit of showing that the CS measurement can be an important matter for schools (Salisbury et al., 1997; Charbonneau and Van Ryzin, 2012). Yet, as Bond and King (2003) argue, these studies often adopted measures of school satisfaction that are specific to the kind of participants (e.g., parents, high school student) and/or of the context under investigation and this hampered their generalizability. Above all, most of these studies estimate school users’ customer satisfaction with instruments designed for more general aims – by selecting from them items that were considered informative, which have not been subjected to systematic psychometric analysis as measures of school users’ customer satisfaction (e.g., Griffith, 1998, 2001; Johnson et al., 2001; Gibbons and Silva, 2011).

This paper means to help to address these limitations. It provides a new instrument of measurement of school users’ satisfaction – QUASUS (QUestionnaire for the Analysis of the School User’s Satisfaction).

### QUASUS

QUASUS is a model – and an associated tool of measurement – of the customer satisfaction which has a general focus, namely it consists of parallel equivalent versions, that can be used with different kinds of users (higher education students, parents, stakeholders) as well as types of school (primary school, secondary school, high school).

QUASUS pays specific attention to the role played by the user’s experience of the relationship with school, considered both a major factor of satisfaction and user’s involvement (Jacobsen et al., 2014). The centrality of the relationship with the user has been highlighted by the Service Management approach (Normann, 1991; Grönroos, 2000). More specifically, the Service Management approach has conceptualized the centrality of the user-provider relationship in the terms of the notion of *prosumership*, which implies a view of the service as an inherent relational event. Prosumership refers to the view of the client-provider relation not only as a source and/or a result of the client’s experience of the service, but as a constitutive component of the service’s construction and delivery. According to this perspective, the client is not the mere user of the service, but both its producer and its consumer – its pro-sumer (Normann, 1991). This means that the user-provider exchange is something more than the mere medium of the functional transaction through which the supply is carried out; it is also a constitutive component of the transaction, one of the main sources of its capacity to generate value.

Service management theory underlines that prosumership is a key point of the provider’s success, given that the very construction of the service depends on the dynamic, co-constructive integration of the client within the boundaries of the process of producing the service. The client-provider relationship in terms of prosumership therefore needs to be taken into account – i.e., to be measured and mapped – not only as the result of the experience of the service, but as one of the components of the service construction.

The centrality of the concept of prosumership may have even higher relevance in the case of school (Mossi and Salvatore, 2012). This is so for two main reasons. First, because of the immateriality of the content of the school service: due to this characteristic, the value that makes up the school action is necessarily generated within and through the dynamic relation between the user and the provider, as a function of the client’s active engagement with the relationship. Second, because of the specificity of the relation between schools and their user; indeed – at least in many countries – the school is not a service provider only, but an institutional body with regards to which users have political rights and duties concerning the participation in setting school policies; therefore it is important to integrate the conceptualization and the measurement of customer satisfaction with specific attention to the way users interpret such a level of commitment to the school service.

In order to model and detect the relational dimension of the customer satisfaction, QUASUS refers to the PROSERV model (Ciavolino et al., 2017, 2018), a general approach to the conceptualization and measurement of the customer satisfaction in the context of service supply. PROSERV integrates the functional and structural facets taken into account also by other models of customer satisfaction with facets concerning the prosumership. More specifically, PROSERV model maps this set of facets in the terms of five dimensions:

1. *Utility* – the evaluation of the service capacity to satisfy the client’s demand.
2. *Process* – the user’s experience of the characteristics of the procedures/actions involved in the provider-user relation – e.g., accessibility and courtesy.
3. *Co-construction* – this is the dimension concerning with prosumership. It concerns the client’s perception of the service as a transaction that adjusts its purposes in order to fit the client’s purpose, thus qualifying as a dynamic of co-construction.
4. *Front-office* – the qualities of the personnel that are the user’s points of contact with the provider – e.g., organizational skills and reliability.
5. *Devices* – the qualities and characteristics of the logistic and structural facets of the service.

QUASUS adjusts the five PROSERV dimensions to the specificity of the school context. In so doing, the QUASUS model is articulated on the following six components:

1. *Teaching-learning process*. It corresponds to the PROSERV *Process*, yet focalized specifically on the didactic exchange substantiating school activities.
2. *Teaching output*. It corresponds to the PROSERV *Utility;* it concerns the quality of the teaching action’s output, namely students’ knowledge, competences and skills.
3. *Educational effectiveness*. This dimension concerns with the PROSERV *Utility* too. Yet, QUASUS considers it separately, following in that a long-standing pedagogical debate (e.g., Salvatore and Scotto di Carlo, 2005), that highlights the opportunity to distinguish two levels/components of the school’s outcome: the building of competence and skills (training) and the promotion of values and adherence to society’s norms (education).
4. *Prosumership*. It corresponds to the PROSERV *Co-construction*; it concerns with the perception of the school’s ability/inclination to modulate its action and boundaries to users’ concerns and demands.
5. *Flexibility*. It corresponds to the PROSERV *Front-office.* It concerns with the flexibility and reliability of the school personnel of direct contact with the user.
6. *Equipment*. It corresponds to the PROSERV *Devices*, focused on logistic and structural aspects of the school activity.

Moreover, similarly to PROSERV, QUASUS model encompasses a separate Overall Satisfaction index, composed of three similar global evaluation of the school (see sub-paragraph *Instrument*). This is so because according to the psycho-social framework QUASUS is based on, the overall satisfaction has to be considered a unique, global, affect-laden connotation of the whole experience of the engagement with the school, rather than an analytic judgment obtained by the linear combination of the evaluation of the single facets of the school action and output. Therefore, QUASUS does not derivate the Overall Satisfaction index from the analytic indexes, but as a separate measure.

## Aims and Hypotheses

The study is aimed at presenting and providing a first validation of the QUASUS. Though the instrument may be applied to all categories of school users (parents, students, stakeholders), the current study focuses on parents. More specifically, the study means to test the following hypotheses:

1. QUASUS proves to be characterized by a good level of reliability (HP1).
2. The six-dimension QUASUS model is able to detect the components comprising the school users’ satisfaction (HP2).
3. Consistently with the interpretation of it as a unique, global connotation of the experience of the school, the 3-item Overall Satisfaction index proves to be a unidimensional measure of the overall satisfaction (HP3).
4. QUASUS dimension Prosumership (i.e., the component characterizing the specificity of the model), is associated significantly with the overall satisfaction, in that showing that it plays a significant role in affecting the user’s attitude toward the school (HP4).

In brief, HP1 concerns with the reliability of the measurement; HP2-HP4 concern with its construct validity.

## Materials and Methods

The hypotheses were tested by means of a two-stage procedure. Firstly (Stage 1), an explorative factorial analysis was performed, in order to check the dimensionality of the instruments preliminarily. Secondly (Stage 2), a confirmatory factorial analysis (CFA) of the dimensions identified in the previous stage was carried out, and with it the reliability of the instrument was estimated again. Moreover, at Stage 2, the relation between the QUASUS dimensions and overall satisfaction was analysed.

Each stage was performed on a specific sample.

### Samples

**FIGURE 1:**
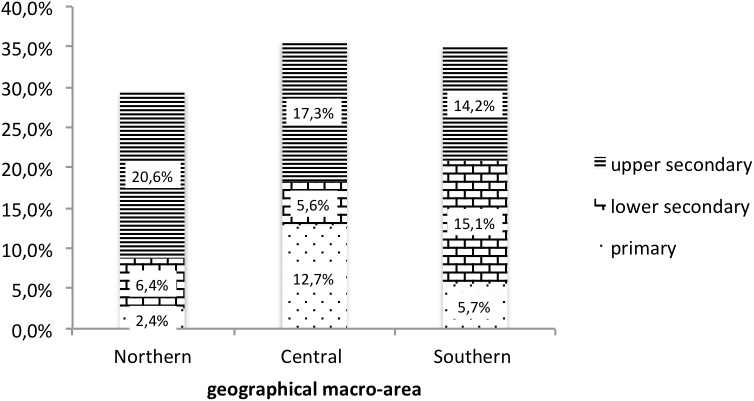
Stage 1 sample. Geographical Area ^∗^ Education.

Stage 1 relied on a convenience cluster sample of parents of students (*N* = 2802) of nine schools located in five Italian regions – three for each geographical macro-area (Northern, Centre and Southern Italy) as well as three for each level (primary, lower secondary, upper secondary). Figure 1 outlines the sample’s distribution as to territories and school levels. It is composed mainly of women (72.5% vs. 50.1% of the Italian population), though it proved to be slightly older than the Italian population (average mean 42.88 [*SD* = 6.21] vs. 40.65 [*SD* = 9.50] for the Italian population). As to education, the sample proved to be underrepresented in the lower level and overrepresented in the higher level, compared to the Italian population (lower secondary: 33.6% vs. 48.9%; upper/post-secondary: 48.8% vs. 38.8%; tertiary education: 17.6% vs. 12.3%).

**FIGURE 2:**
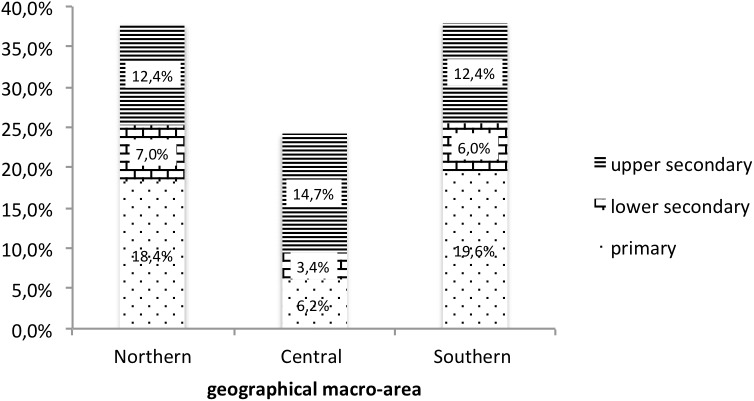
Stage 2 sample. Geographical Area ^∗^ Education.

Stage 2 was based on a convenience cluster sample of 16 Italian schools (*N* = 1365). The schools were selected so that each of the three macro-regions of Italy (Northern, Centre, Southern) were represented by at least three schools, one for each level (primary, lower secondary, upper secondary) – cf. Figure 2.

This second sample is also characterized by a higher proportion of women compared to the Italian population (70.1% vs. 50.1%) and a slightly higher age (41.8 [*SD* = 6.6]) similar to that of the Italian population (40.9 [*SD* = 9.5]). The sample was marked by a similar level of education to that of the Italian population – lower secondary: 48.2% (Italian population: 48.9%); upper secondary and post-secondary, non-tertiary 40.5% (Italian population: 38.8%); tertiary education: 11.4% (Italian population: 12.3%).

### Instruments

QUASUS is composed of two parts.

The first part comprises 35 items, each of them aimed at measuring the user’s satisfaction of a specific facet/characteristic of the school (henceforth we refer to these items as *analytic judgments*). These items are associated to a 6-point Likert-like scale, ranging from “extremely dissatisfied” to “extremely satisfied”. No intermediate point is introduced, in order to ask respondents to position themselves between positive versus negative judgments. The 35 items were selected from a broader set of items (*n* = 80), defined on the basis of a review of the literature and preliminary explorative focus groups with parents of several Italian schools. The original set was constructed in order to take all relevant facets and components of school into account.

The selection of items was carried following four main criteria: (a) to make the number of items as low as possible; (b) to prevent response-set; (c) to optimize the instrument’s inner consistency; (d) to increase the interpretability of items, by keeping only those that had clear, mono-dimensional content. To this end, a series of preliminary explorative factorial analysis were performed, which led to the definition of the final set of 35 items.

The second part of the questionnaire was composed of three items assessing the overall satisfaction. The three items are associated to the same 6-point Likert-like scale used in the first part (from “extremely dissatisfied” to “extremely satisfied”). Henceforth we refer to these items as *overall judgments*. The three items have similar content and are aggregated in a single index of Overall Satisfaction. The choice of using three similar items is designed to increase the reliability of the estimation. It is worth highlighting that, unlike what is suggested by the disconfirmation paradigm (Yüksel and Yüksel, 2008), in the current work we use overall satisfaction as a separate, independent index (rather than calculating it by combining the score of satisfaction associated with the various attributes of the service). This was done in order to take the qualitative difference between the overall satisfaction and the satisfaction obtained from the aggregation of the analytic satisfaction items, each of which take a specific facet into account. Indeed, according to the psycho-social model QUASUS is grounded on, overall satisfaction is an affect-laden, generalized connotation of the whole experience of relation with the school, rather than a content-based, functional judgment, as in each of the 35 items involved (Mossi and Salvatore, 2012; Ciavolino et al., 2017). Accordingly, we do not consider it as directly derivable from the linear combination of the 35 analytic judgments.

Finally, the QUASUS instrument was integrated with a set of indicators aimed at collecting information on respondents’ socio-demographic characteristics. QUASUS is reported in Supplementary Annex 1.

The questionnaire held information as to the aims of the research, modality of data treatment as well as the request to participate voluntarily to it. Consistently with the ethical code of the Italian Psychology Association (AIP)^1^ and the Italian Code on the protection of personal data (Legislative decree No 196/2003), the return of questionnaire filled by the participant to the research team has been considered as informed consent. An ethics approval was not required for this research as per your Institution’s guidelines and national regulations.

### Data Analysis

At Stage 1, the reliability of the instrument (split-half analyses) and its inner consistency (Cronbach’s alpha) was estimated (HP1).

Moreover, a first, preliminary stream of analyses of the dimensionality of the instrument was performed by means of an exploratory factorial analysis – Principal Axis Factoring (PAF) – Promax rotation. The aim of this preliminary PAFs was to detect the subset of items allegedly substantiating the QUASUS model’s dimensions to be subjected to the Stage 2 confirmatory analysis.

More specifically, the PAF procedure was applied to the 35 analytic judgments (HP2) and three items concerning overall satisfaction (HP3) separately. All PAF procedures used Listwise detection in order to processing missing values and were elaborated through the SPSS 22 package. The number of factors to select was based on eigenvalues and Catell’s Screen Test.

At Stage 2, the following paths of analysis were performed.

First, an analysis was made of the perceived equidistance between the items’ Likert-points. This analysis is relevant because the procedure of data analysis adopted assumes that points of the Likert scales (from extremely dissatisfied to extremely satisfied) are perceived by respondents: (a) as measuring a single continuous dimension, (b) equidistant from each other. The estimation of the perception of equidistance was carried out by means of the procedure suggested by Marradi (1981), based on Simple Correspondence Analysis – ACS (Fisher, 1925). The ACS allows the evaluation of the relationship between two nominal variables describing the association between the categories of both variables. More specifically, one output of the ACS is the estimation of the independence among the modalities of one variable compared to those of the other variable. This estimation can be detected in geometric terms, by means of the projection of the points representing the categories of the variable on the factorial space describing the relation among the two variables. In the case of the current analysis, the ACS was applied to the matrix defined by 35 items measuring the parent’s satisfaction (i)^∗^ Likert-points (j). Each ij-th cell of the matrix held the frequency of the i-th item on the j-th Likert-point. Analysis focused on the column profile, namely on the association among the Likert-like points. In so doing, the projection of these points on the 2-dimension factorial space (due to the composition of the matrix, the first factor concerns the response value, while the second factor represents the intensity of the expression of attitude) graphically detected the distance between the points, in turn interpretable as the marker of how the relation (i.e., the distance) among Likert-points was perceived by respondents.

Second, two procedures of CFA (Joreskog et al., 1979) were carried out. One procedure was applied on the 35 analytic judgments and aimed at testing the dimensions extracted by the previous procedure of explorative factorial analysis (HP2). The dimensions obtained from the Stage 1 exploratory analyses were introduced as latent variable in the structural model subjected to testing. Each latent variable was measured initially by means of the subset of items associated with the corresponding dimension of the Stage 1 exploratory factorial analysis. The second CFA model was applied on the three overall satisfaction items in order to estimate the Overall index (HP3). In both cases, given that skewness and kurtosis indexes of all items were lower than the standard threshold (1.00), maximum likelihood estimation-ML was used in order to estimate the model’s goodness of fit (Muthén and Kaplan, 1985).

Following the suggestion of Fornell and Larcker (1981), in order to evaluate the effectiveness of the confirmatory factor analysis measurement model, besides the usual significance indexes, further reliability indexes of factorial dimensions were estimated: the reliability of the individual indicators, the explained mean variance (AVE) and the Composite Reliability of the construct (CR or omega). The analysis was performed by means of the MPlus7 package.

Third, the analysis of the reliability and inner consistency of both the whole set of 35 analytic judgments and of the single scales was performed (HP1). To this end, split-half indexes (Guttman Split-Half coefficient and Spearman–Brown Coefficient for Unequal Length) and Cronbach’s alpha were also computed on sample 2. Moreover, following Cortina (1993), Average Inter-item Correlations and Alpha’s standard error (S.E.) were calculated for each construct evaluated. These two additional indexes were considered because of their utility in evaluating the mono-dimensionality of the constructs (Schmitt, 1996).

Finally, in order to test Hypothesis 4, the association between Prosumership and Overall satisfaction was estimated by means of Pearson’s coefficient of correlation.

## Results

### Stage 1

#### Reliability Coefficients

All measures of reliability of the QUASUS 35 items proved to be high: Guttman’s Split-Half coefficient: 0.918; Spearman–Brown Coefficient for Unequal Length: 0.918; Cronbach’s alpha: 0.951.

Cronbach’s alpha of the three overall satisfaction items was 0.90.

#### Exploratory Factorial Analyses

The PAF applied on the 35-item block proved an adequate sampling adequacy: Bartlett’s test of sphericity was significant at the <0.001 level and the Kaiser–Meyer–Olkin Measure (KMO) of sampling adequacy was high (0.97). Four factors were selected. Once corrected the estimation in reason of the oblique rotation, these factors explained 46.5% of the variance (cf. Table 1).

**Table 1:** Exploratory factor analysis – Stage 1. Factors extracted.

	% of Variance – corrected by oblique rotation^∗^	Cumulative %
**Principal Axis Factoring on the whole dataset (35 item)**		
Factor 1	19.24	19.24
Factor 2	10.32	29.56
Factor 3	8.99	38.55
Factor 4	7.96	46.51
**Principal Axis Factoring on the 14 item block corresponding to the first PAF’s Factor 1**		
Factor 1a	22.66	22.66
Factor 1b	14.58	37.24
Factor 1c	13.31	50.54

*^∗^Promax rotation. Estimation corrected for the oblique rotation.*

The first factor proved to be comprised of 14 items concerning with both school process and outcome, therefore marking an overarching, *global educational dimension* (cf. Table 2). Given the relevance that the QUASUS model recognizes of the distinction between process and outcome and between education and training components of the outcome (cf. *Introduction*), we decided to carry out a second PAF on the 14-item block, in order to verify if this block should prove to be articulated in sub-dimensions, once considered separately from the whole dataset. We did so with the purpose of optimizing the identification of the factorial structure to be subjected to the Stage 2 confirmatory analysis (for the rationale of this procedure, see Tabachnick and Fidell, 2007; Hair et al., 2010; Gaskin, 2016).

**Table 2:** Composition and factor loadings of the QUASUS’s scales – Stage 1.

Scale	n°	Item	1	2	3	4
**Factorial analysis A**						
Global Education	14	S01 correct recognition for “education commitment”	0.73	0.02	−0.01	−0.01
Dimension		S02 quantity and difficulty of homework assignments	0.42	0.02	−0.02	0.19
		S03 ability to adapt the lesson to the level of the students	0.71	−0.09	0.03	0.05
		S04 credibility of the results achieved by the student	0.79	−0.02	−0.07	0.01
		S05 commitment of the teaching staff	0.79	−0.07	−0.11	0.13
		S06 information on the progress and the difficulties of the student	0.58	−0.07	0.08	0.11
		S07 collaboration among teachers	0.58	0.00	0.04	0.16
		S08 promotion of values	0.63	0.03	0.06	−0.02
		S09 promoting collaboration with peers	0.66	0.02	0.11	−0.14
		S10 training to respect the environment	0.38	0.14	0.22	0.02
		S11 attention to the social context	0.48	0.08	0.13	0.06
		S12 development of the autonomy of the pupils	0.65	0.07	0.01	0.01
		S13 development of pupils’ personal skills	0.69	0.08	0.08	−0.10
		S14 development of critical thinking skills of pupils	0.72	0.00	−0.08	0.00
Equipment	8	S15 maintenance and state of conservation of school buildings	0.01	0.77	−0.04	−0.04
		S16 quality of the logistic structures	−0.08	0.69	0.01	0.10
		S17 IT equipment and scientific disciplines	0.07	0.62	−0.06	0.01
		S18 aesthetic quality of the premises	−0.06	0.71	0.01	0.00
		S19 dimensions and equipment of the gym	0.06	0.60	−0.15	0.04
		S20 existence of external spaces usable by the pupils	0.03	0.55	−0.02	0.07
		S21 quality of furnishings	−0.08	0.65	0.15	−0.04
		S22 disabled facilities and services	0.06	0.54	0.03	−0.03
Prosumership	6	S23 family participation in the school initiatives	0.01	−0.08	0.88	−0.06
		S24 inclusion of parents in school initiatives	0.01	−0.03	0.77	0.01
		S25 consideration of the opinions and proposals of the parents	0.17	0.04	0.53	0.03
		S26 dissemination initiatives aimed at pupils and parents	0.18	0.06	0.55	−0.01
		S27 existence of channels to make complaints	0.15	0.18	0.46	−0.01
		S28 flexibility of schedules according to the commitments of the parents	−0.01	−0.05	0.43	0.32
Flexibility	7	S29 respect to the opening hours for the public	0.00	−0.01	0.09	0.59
		S30 head teacher’s commitment	0.08	0.09	−0.11	0.70
		S31 secretarial services	0.03	0.08	0.08	0.53
		S32 availability of the principal	0.03	0.09	0.01	0.62
		S33 distance from the town	0.15	−0.01	−0.09	0.42
		S34 availability in the reception hours	0.30	−0.09	0.03	0.37
		S35 compatibility between school and parents’ schedules	0.01	−0.05	0.35	0.37
	Eigenvalue		13.57	2.33	1.25	1.21
	Factor Correlation Matrix		1	2	3	4
	1		1.000	0.575	0.748	0.734
	2		0.575	1.000	0.618	0.607
	3		0.748	0.618	1.000	0.685
	4		0.734	0.607	0.685	1.000
**Factorial analysis B**						
Teaching-learning Process	7	S01 correct recognition for “education commitment”	0.65	−0.03	0.15	
		S02 quantity and difficulty of homework assignments	0.62	−0.04	0.00	
		S03 ability to adapt the lesson to the level of the students	0.62	0.18	−0.05	
		S04 credibility of the results achieved by the student	0.52	−0.01	0.26	
		S05 commitment of the teaching staff	0.55	0.10	0.16	
		S06 information on the progress and the difficulties of the student	0.54	0.13	0.05	
		S07 collaboration among teachers	0.52	0.27	−0.01	
Educational Effectiveness	4	S08 promotion of values	−0.01	0.70	0.08	
		S09 promoting collaboration with peers	0.10	0.54	0.06	
		S10 training to respect the environment	0.05	0.45	0.20	
		S11 attention to the social context	0.21	0.41	0.08	
Teaching Output	3	S12 development of the autonomy of the pupils	0.03	0.10	0.65	
		S13 development of pupils’ personal skills	0.09	0.11	0.58	
		S14 development of critical thinking skills of pupils	0.11	0.11	0.48	
	Eigenvalue		7.14	0.78	0.67	
	Factor Correlation Matrix		1	2	3	
	1		1.000	0.809	0.811	
	2		0.809	1.000	0.802	
	3		0.811	0.802	1.000	

Also the PAF applied on the 14-item block showed sampling adequacy – Bartlett’s test of sphericity *p* < 0.001; the Kaiser–Meyer–Olkin Measure (KMO): 0.97. Three factors were selected. Once corrected the estimation in reason of the oblique rotation, these factors explained 50.54% of the variance (cf. Table 1).

Combining the results of both factorial analyses (i.e., the PFA on the 35 items and the PFA on the 14-item block), we identified 6 dimensions (3 dimensions + 3 sub-dimensions).

Finally, the exploratory factor analysis applied to the three overall items proved the mono-dimensionality of that scale – one factor was extracted, which explained 83.49% of variability.

### Stage 2

#### Perceived Equidistance Among Likert-Points

**FIGURE 3:**
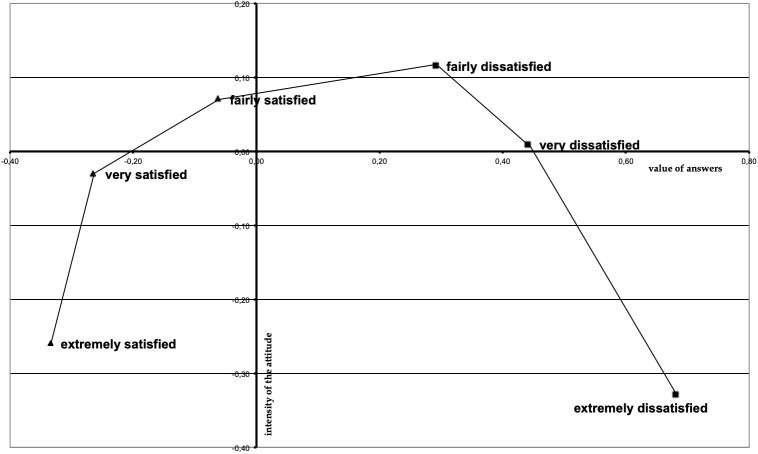
Perception of the distance between categories of satisfaction scale.

Figure 3 outlines the output of the Correspondence Analysis applied for the sake of estimating the perceived distance between the Likert points. The matrix was based on *N* = 1365 respondents. As one can see, there is substantial equidistance between the six response categories used – with the partial exception of the category “extremely dissatisfied”, which is used as indicative of an even more extreme negative judgment. From a complementary standpoint, one can see the almost central position of the category “fairly satisfied”. This means that respondents tended to use this category as an intermediate point, probably due to the absence of a neutral point equidistant from the two poles of the scale.

#### Confirmatory Factor Analysis

The CFA applied on the 35 analytic judgements was performed on a subsample of *N* = 1183 respondents, given that *N* = 182 respondents had to be excluded because listwise deletion of missing data patterns.

**FIGURE 4:**
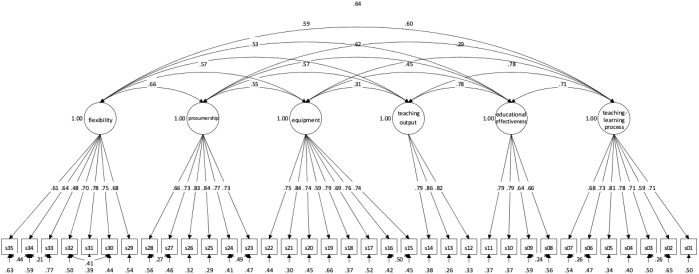
Stage 2: Confirmatory Factorial Analysis (CFA). Structural model’s Standardized Coefficients. *s01–s35* denotes QUASUS analytic judgments of satisfaction as reported in Table 2.

The model that was tested is based on the Stage 1 PFAs output. The model proved to fit with data (Chi-Square Value = 2449.960; d.f. 536; RMSEA = 0.051 (90% C.I.: 0.049–0.053; probability <= 0.05 0.177); TLI = 0.93; CFI = 0.93; SRMR = 0.049). Figure 4 outlines the Model.

All items have a relationship with the expected latent variable only. λ coefficients proved to be quite high and similar among them – they range from 0.48 to 0.86.

The second CFA was applied to the three overall satisfaction items. This analysis was performed on a subsample of *N* = 1323 respondents from the general sample used in stage 2, given that *N* = 42 respondents had to be excluded because of missing on all responses.

**FIGURE 5:**
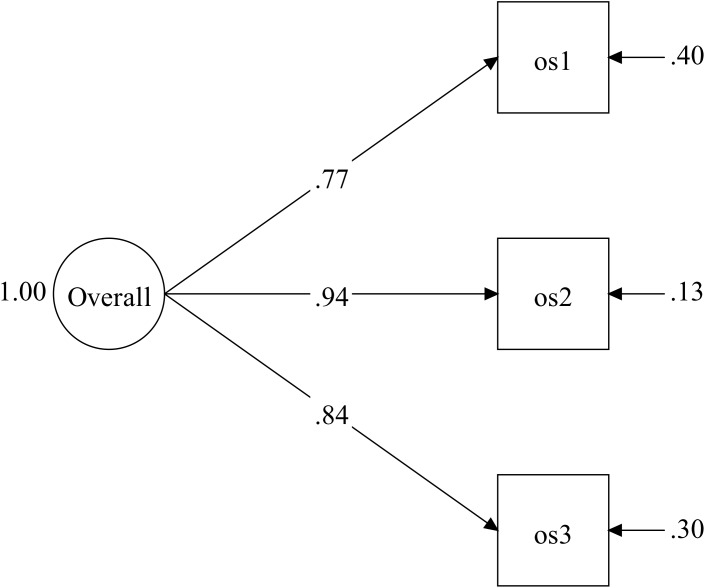
Stage 2: CFA of Overall Satisfaction: Standardized Coefficients. *os(n):* Overall Satisfaction items (cf. Supplementary Annex 1).

Indexes of fitness of the model proved to be adequate (TLI = 1, CFI = 1, SRMR = 0), and the three λ coefficients of the manifest variables proved to be significant (*p*-value < 0.001; cf. Figure 5). λ coefficients range from 0.77 to 0.94.

In Table 3 measures of both CFA power for the measurement model for each latent variable (Fornell and Larcker, 1981) are reported. In general, indexes proved higher values than the cut-off suggested by the literature (reliability of the individual indicators ≥ 0.30; AVE ≥ 0.50; Composite Reliability ≥ 0.60). Only for the flexibility scale the values showed a lower, but still significant, level.

**Table 3:** Reliability of the latent variables (both CFAs).

	Reliability of the individual indicator (min – max)		Average Variance Extracted (AVE)	Composite Reliability (CR) or Omega
**First CFA (35 analytic judgment items)**				
Sat 1 – Teaching-learning Process	0.35	0.66	0.51	0.88
Sat 2 – Educational Effectiveness	0.41	0.63	0.53	0.81
Sat 3 – Teaching output	0.62	0.74	0.68	0.86
Sat 4 – Equipment	0.34	0.70	0.55	0.91
Sat 5 – Prosumership	0.44	0.71	0.58	0.89
Sat 6 – Flexibility	0.28	0.61	0.45	0.85
**Second CFA (3 overall satisfaction items)**				
Overall	0.60	0.87	0.72	0.89

#### Reliability Coefficients

Stage 2 reliability analyses confirmed the levels of reliability obtained at Stage 1.

Table 4 summarises the output of the analysis of reliability (Guttman and Spearman–Brown split-half coefficients) and inner consistency (Cronbach’s alpha, mean of Inter-Item Correlations and Standard Error di Alpha).

**Table 4:** Cronbach’s alphas and Split-Half coefficients for satisfaction and overall evaluation.

	*N*^∧^ of item	Split/half Guttman	Split/half S-B	Alpha Cronbach	Mean of Inter-Item Corr.	α S.E.
Whole set of items	35	0.863	0.863	0.948	0.350	0.005
Scale 1 – Teaching-learning process	7	0.814	0.828	0.885	0.525	0.016
Scale 2 – Educational Effectiveness	4	0.757	0.757	0.816	0.528	0.021
Scale 3 – Teaching output	3	–	–	0.857	0.667	0.038
Scale 4 – Equipment	8	0.858	0.858	0.906	0.556	0.017
Scale 5 – Prosumership	6	0.839	0.839	0.899	0.599	0.023
Scale 6 – Flexibility	7	0.725	0.739	0.858	0.469	0.024
Overall Satisfaction	3	–	–	0.885	0.720	0.038

As to the whole set of 35 analytical judgments, the reliability coefficients reached satisfactory values: Guttman Split-Half coefficient = 0.863; Spearman–Brown Coefficient for Unequal Length = 0.863; Cronbach’s Alpha = 0.948, average Inter-item Correlation = 0.350; standard error of Alpha = 0.005. As to the six scales separately, with the exception of the Guttman and Spearman–Brown coefficient (both 0.757) of Scale 2 (Educational Effectiveness) and Guttman (0.725) and Spearman–Brown coefficient (0.739) of Scale 5 (Flexibility), no split-half coefficient was lower than 0.800; Cronbach’s Alpha ranged from 0.816 to 0.906 and standard error of Alpha was always less than 0.040.

Also Cronbach’s alpha of the 3-item measuring the overall satisfaction proved to have high inner consistency – Alpha value was 0.885; inter-item correlation was 0.720; the standard error of Alpha was 0.038 (split half was not estimated because of the small number of items).

#### Prosumership and Global Satisfaction

In order to test the third hypothesis, the coefficient of correlation between the prosumership scale and overall satisfaction was calculated: *r* = 0.448 (*p* < 0.01). For the sake of comparison, correlations between overall satisfaction and the other dimensions that emerged from the CFA applied on the 35 items of satisfaction are reported too. All coefficients proved to be quite robust and significant (cf. Table 5).

**Table 5:** Correlation between overall satisfaction and QUASUS’s scales.

	Educational Effectiveness	Teaching output	Teaching-learning process	Equipment	Flexibility	Prosumership
Overall satisfaction	0.502^∗∗^	0.489^∗∗^	0.519^∗∗^	0.331^∗∗^	0.479^∗∗^	0.448^∗∗^

*^∗∗^ Correlation is significant at the 0.01 level (2-tailed).*

Teaching-learning process (*r* = 0.519) is the factor with the highest correlation with overall satisfaction, followed by Educational effectiveness (*r* = 0.502), Teaching output (*r* = 0.489) and Flexibility (*r* = 0.479) with similar coefficients. Equipment (*r* = 0.331) proved to be the dimension least associated with global satisfaction.

## General Discussion

This article outlined the conceptual framework and the first evidence supporting the validity of QUASUS, a new tool for measuring and analysing the satisfaction of school users.

Findings are consistent with the three hypotheses that were tested.

As to the first hypothesis – i.e., the reliability of the instrument – in both the stage of analysis, based on different samples, reliability indexes proved to be significant and at least quite high. Moreover, the perceived distance between the points of the Likert-scale proved to be quite balanced. Therefore, judgments of satisfaction obtained can be considered quite an unbiased evaluation of satisfaction.

As to the second hypothesis, the combination of preliminary explorative analyses and confirmatory analysis provided evidence in support of the 6-dimension QUASUS model’s construct validity Indeed, the instrument proved to have a stable 6-factor structure. The main source of variance of the parents’ satisfaction is associated with facets concerning the process and the outcome of the school activities – i.e., the dimensions: *Educational Effectiveness*, *Teaching-learning Process* and *Teaching output*. However, the instrument proved to be able to detect a component concerning the *Prosumership*, which represents as a specific dimension of the factorial structure, explaining a non-marginal proportion of variance (8.99%), in addition to the dimensions of *Flexibility* and *Equipment*.

The comparison between the explorative and the confirmatory analyses makes further two considerations worth being added. First, it has to be noted that the first exploratory analysis has identified a global educational dimension as first factor. Accordingly, the three dimensions concerning the process (*Teaching-Learning process*) and the outcome (*Educational effectiveness* and *Teaching output*) which were supported by the confirmatory analysis eventually, represent sub-dimensions of this global educational dimension (as obtained by the second explorative factorial analysis). This means that even if the three dimensions are worth being distinguished – as the confirmatory analysis attests – however, parents tend to consider and enact them in convergent way, as if they were articulation of an unique overarching domain of meaning. Such a result was not foreseen by the current study’s hypotheses; yet it is not inconsistent with the QUASUS model. Indeed, the finding highlights that given the parents’ not direct involvement, their perception of the school’s teaching-learning action is considered globally in first instance, firstly in terms/through the lens of the daily interaction between teachers and students (as shown by the fact that the main sub-dimension of the educational generalized dimension is *Teaching-learning process*). In other words, parents tend to merge process and outcome, because of the easer representability of the former, which for this reason is used as the marker of the latter too. Incidentally, this interpretation is consistent with the strategic relevance that the Service Management theory attributes to the dynamic of contact between user and providers’ human resources, considered as the “moment of truth” where the perception of the service value is built (Normann, 1991). As highlighted by this conceptual framework – in a way that is fully consistent with the specificity of the school context – the relevance of the moment of truth is a direct consequence of the immateriality of the service; due to this characteristic, the user has less chance to anchor her/his evaluation on the concrete aspects of the output and therefore she/he needs to foreground the experience of the process of interaction with it (Ciavolino et al., 2018).

Second, it has to be highlighted that, though as sub-dimension, analyses have confirmed the QUASUS model’s distinction between two levels of outcome – the educational level (i.e., *Educational effectiveness*) and the training/didactic level (i.e., *Teaching output*). This articulation is the main specificity of the QUASUS model with respect to the PROSERV (Ciavolino et al., 2018) – which is a general approach aimed at modelling and measuring the customer satisfaction in the context of services – introduced in order to take into account the peculiarity of the school context. On the other hand, one has to recognize that the distinction/dialectics between education and training might not be universally relevant – indeed, whereas it is foregrounded by the continental European pedagogical approaches (due to the influence of the idealistic tradition of thought), it may be less relevant in pragmatic Anglo-Saxon contexts (Massa, 1997).

As to the third hypothesis, it is consistent with findings too – the Overall Satisfaction index resulted to have a unidimensional structure, both in the exploratory and confirmatory analyses. This means that the global satisfaction has to be considered an overarching affect-laden feeling, which connotes the experience of relation with the school as a whole. Consistently with the psychological theory that highlights the homogenizing valence of the affects (e.g., Salvatore and Freda, 2011; Tonti and Salvatore, 2015; Salvatore et al., 2018), the semantic differences among the three items composing the index are backgrounded, being rather considered by the respondents as equivalent expression of the generalized attitude toward the school.

Finally, as expected by the Hypothesis 4, the Prosumership proved to be associated with the overall satisfaction, in that showing that the satisfaction with the school’s capacity to involve parents in the design and supply of school activities helps to foster the global relationship between parents and school, which affects overall satisfaction.

What interests us here is to highlight the fact that the application of QUASUS can offer clues on how to map dynamically and contribute to a better understanding of the school-family relationship, a purpose that goes quite beyond the mere recognition of the level of satisfaction. According to this perspective, satisfaction is the mean, not the goal of the analysis. In other words, the analysis of satisfaction should not be conceived of as an operation aimed at collecting an objective judgment on school’s activity, but as an index to be further interpreted in order to better understand the dynamics of the relationship between school and users.

To this end, the role of prosumership – i.e., the parents’ expectation of participating actively in shaping the school’s operation as partners, rather than mere users – highlighted by the current study is worth considering. Indeed, the relevance of prosumership means that the promotion of the school-family relationship is not only a matter of improving the technical core of the school action (i.e., the education factors mapped by QUASUS dimensions of satisfaction such as *Educational effectiveness, Teaching-Learning Process* and *Teaching output*), but is also the ability to build a relationship with users in terms of service.

In sum, the tenet of prosumership makes the relation of partnership with parents a key point of the school’s success. According to the prosumership view, the quality of the school action depends on the dynamic, co-constructive integration of the client within the school’s boundaries – i.e., in the school’s capacity to actively involve parents and conversely to adjust to this weakening of its boundaries.

Before concluding, it is worth highlighting limitations to the current study. Firstly, it does not consider the evolution of constructs over time; longitudinal studies are necessary to investigate and clarify the relationships between the variables over time. The use of self-report data represents a further major limitation. Indeed, this approach suffers from well-known limits. The analysis of the perceived distance among the Likert points is only a partial remedy to this limit. In this study the differential effects that might be due to gender, education, age as well as specific users’ demand (e.g., parents with students receiving special education service or disadvantaged students), were not investigated. On the other hand, these aspects can play an even relevant role in affecting the level and the structure of the user’s satisfaction. Therefore, further studies are required to estimate these alleged effects. Finally, it has to be recognized that the findings lack generalizability. Although the size of the samples was large, the study was limited to the Italian context. Further research is therefore needed in order to extend the arguments and conclusions reported above to other socio-cultural and institutional contexts.

## Author Contributions

PM and SS conceived and designed the study. MT organized the database. PM performed the statistical analysis. EI wrote the first draft of the manuscript. PM, EI, and SS wrote sections of the manuscript. All authors contributed to manuscript revision, read and approved the submitted version.

## Conflict of Interest Statement

The authors declare that the research was conducted in the absence of any commercial or financial relationships that could be construed as a potential conflict of interest.

## Footnotes

1 http://www.aipass.org/node/11560
