# Supplementary material for: QUASUS: A Tool for Measuring the Parents’ School Satisfaction
Source: Front Psychol. 2019 Jan 22;10:13. doi: 10.3389/fpsyg.2019.00013 (PMC6349731; doi:10.3389/fpsyg.2019.00013)
Supplement: Supplementary file 1 [file Table_1.DOC]

Annex 1. QUASUS items

| ☹  ☹  ☹ | ☹  ☹ | ☹ | ☺ | ☺  ☺ | ☺  ☺  ☺ |
| --- | --- | --- | --- | --- | --- |
| **Extremely dissatisfied** | **Very dissatisfied** | **Quite dissatisfied** | **Quite**  **satisfied** | **Very**  **satisfied** | **Extremely**  **satisfied** |

|  |  | ☹  ☹  ☹ | ☹  ☹ | ☹ | ☺ | ☺  ☺ | ☺  ☺  ☺ |
| --- | --- | --- | --- | --- | --- | --- | --- |
| S01 | correct recognition for 'education commitment’ | ❑ | ❑ | ❑ | ❑ | ❑ | ❑ |
| S02 | quantity and difficulty of homework assignments | ❑ | ❑ | ❑ | ❑ | ❑ | ❑ |
| S03 | ability to adapt the lesson to the level of the students | ❑ | ❑ | ❑ | ❑ | ❑ | ❑ |
| S04 | credibility of the results achieved by the student | ❑ | ❑ | ❑ | ❑ | ❑ | ❑ |
| S05 | commitment of the teaching staff | ❑ | ❑ | ❑ | ❑ | ❑ | ❑ |
| S06 | information on the progress and the difficulties of the student | ❑ | ❑ | ❑ | ❑ | ❑ | ❑ |
| S07 | collaboration among teachers | ❑ | ❑ | ❑ | ❑ | ❑ | ❑ |
| S08 | promotion of values | ❑ | ❑ | ❑ | ❑ | ❑ | ❑ |
| S09 | promoting collaboration with peers | ❑ | ❑ | ❑ | ❑ | ❑ | ❑ |
| S10 | training to respect the environment | ❑ | ❑ | ❑ | ❑ | ❑ | ❑ |
| S11 | attention to the social context | ❑ | ❑ | ❑ | ❑ | ❑ | ❑ |
| S12 | development of the autonomy of the pupils | ❑ | ❑ | ❑ | ❑ | ❑ | ❑ |
| S13 | development of pupils' personal skills | ❑ | ❑ | ❑ | ❑ | ❑ | ❑ |
| S14 | development of critical thinking skills of pupils | ❑ | ❑ | ❑ | ❑ | ❑ | ❑ |
| S15 | maintenance and state of conservation of school buildings | ❑ | ❑ | ❑ | ❑ | ❑ | ❑ |
| S16 | quality of the logistic structures | ❑ | ❑ | ❑ | ❑ | ❑ | ❑ |
| S17 | IT equipment and scientific disciplines | ❑ | ❑ | ❑ | ❑ | ❑ | ❑ |
| S18 | aesthetic quality of the premises | ❑ | ❑ | ❑ | ❑ | ❑ | ❑ |
| S19 | dimensions and equipment of the gym | ❑ | ❑ | ❑ | ❑ | ❑ | ❑ |
| S20 | existence of external spaces usable by the pupils | ❑ | ❑ | ❑ | ❑ | ❑ | ❑ |
| S21 | quality of furnishings | ❑ | ❑ | ❑ | ❑ | ❑ | ❑ |
| S22 | disabled facilities and services | ❑ | ❑ | ❑ | ❑ | ❑ | ❑ |
| S23 | family participation in the school initiatives | ❑ | ❑ | ❑ | ❑ | ❑ | ❑ |
| S24 | inclusion of parents in school initiatives | ❑ | ❑ | ❑ | ❑ | ❑ | ❑ |
| S25 | consideration of the opinions and proposals of the parents | ❑ | ❑ | ❑ | ❑ | ❑ | ❑ |
| S26 | dissemination initiatives aimed at pupils and parents | ❑ | ❑ | ❑ | ❑ | ❑ | ❑ |
| S27 | existence of channels to make complaints | ❑ | ❑ | ❑ | ❑ | ❑ | ❑ |
| S28 | flexibility of schedules according to the commitments of the parents | ❑ | ❑ | ❑ | ❑ | ❑ | ❑ |
| S29 | respect to the opening hours for the public | ❑ | ❑ | ❑ | ❑ | ❑ | ❑ |
| S30 | head teacher's commitment | ❑ | ❑ | ❑ | ❑ | ❑ | ❑ |
| S31 | secretarial services | ❑ | ❑ | ❑ | ❑ | ❑ | ❑ |
| S32 | availability of the principal | ❑ | ❑ | ❑ | ❑ | ❑ | ❑ |
| S33 | distance from the town | ❑ | ❑ | ❑ | ❑ | ❑ | ❑ |
| S34 | availability in the reception hours | ❑ | ❑ | ❑ | ❑ | ❑ | ❑ |
| S35 | compatibility between school and parents' schedules | ❑ | ❑ | ❑ | ❑ | ❑ | ❑ |

|  |  | ☹  ☹  ☹ | ☹  ☹ | ☹ | ☺ | ☺  ☺ | ☺  ☺  ☺ |
| --- | --- | --- | --- | --- | --- | --- | --- |
| os1 | What is your degree of satisfaction with how the school carries on its activities? | **Extremely dissatisfied** | **Very dissatisfied** | **Quite dissatisfied** | **Quite**  **satisfied** | **Very satisfied** | **Extremely satisfied** |

|  |  | ☹  ☹  ☹ | ☹  ☹ | ☹ | ☺ | ☺  ☺ | ☺  ☺  ☺ |
| --- | --- | --- | --- | --- | --- | --- | --- |
| os2 | Is the overall quality of your child's school in your opinion? | **Extremely dissatisfied** | **Very dissatisfied** | **Quite dissatisfied** | **Quite**  **satisfied** | **Very satisfied** | **Extremely satisfied** |

|  |  | ☹  ☹  ☹ | ☹  ☹ | ☹ | ☺ | ☺  ☺ | ☺  ☺  ☺ |
| --- | --- | --- | --- | --- | --- | --- | --- |
| os3 | Do your child's school make you? | **Extremely dissatisfied** | **Very dissatisfied** | **Quite dissatisfied** | **Quite**  **satisfied** | **Very satisfied** | **Extremely satisfied** |
